# A Real-Time Detection Method for BDS Signal in Space Anomalies

**DOI:** 10.3390/s19061437

**Published:** 2019-03-23

**Authors:** Chun Cheng, Yuxin Zhao, Liang Li, Lin Zhao

**Affiliations:** College of Automation, Harbin Engineering University, Harbin 150001, China; chengchun2@hrbeu.edu.cn (C.C.); zhaoyuxin@hrbeu.edu.cn (Y.Z.); zhaolin@hrbeu.edu.cn (L.Z.)

**Keywords:** BeiDou navigation satellite system (BDS), Signal In Space (SIS), anomaly detection, integrity

## Abstract

Signal In Space (SIS) anomalies in satellite navigation systems can degrade satellite-based navigation and positioning performance. The occurrence of SIS anomalies from the BeiDou navigation satellite System (BDS) may be more frequent than for the Global Positioning System (GPS). In order to guarantee the integrity of BDS users, detecting and excluding SIS anomalies is indispensable. The traditional method through the comparison between the final precision ephemeris and the broadcast ephemeris is limited by the issue of long latency of precision ephemeris release. Through the statistical characteristics analysis of Signal In Space User Range Error (SISURE), we propose a real-time Instantaneous SISURE (IURE) estimation method by using the Kalman filtering-based carrier-smoothed-code to detect and exclude BDS SIS anomalies, in which the threshold for BDS IURE anomaly detection are obtained from the integrity requirement. The experimental results based on 1 Hz data from ground observations show that the proposed method has an estimation accuracy of 1.1 m for BDS IURE. The test results show that the proposed method can effectively detect the SIS anomalies caused by either orbit faults or clock faults.

## 1. Introduction

The BeiDou navigation satellite System (BDS) will provide Positioning, Navigation and Timing (PNT) services for the Asia-Pacific region before 2020 [1]. For most of real-time PNT users, the orbit and clock parameters are derived from broadcast ephemeris. In order to ensure the reliability of positioning, the health flag contained in the broadcast ephemeris is widely used to indicate the availability of satellites [2]. However, the broadcast ephemeris belongs to the predicted ephemeris. Due to the possible inconsistency between the prediction model and the true kinetic model, the predicted ephemeris sometimes cannot accurately describe the true satellite orbit and clock, and cause largely biased position errors. Therefore, real-time detection of satellite orbit and clock anomaly is indispensable for the reliability of positioning.

Signal In Space (SIS) errors are mainly composed of satellite orbit and clock errors, which are the major error sources of ranging observations [3,4,5]. Signal In Space User Range Error (SISURE) is the projection of SIS errors on the direction of line-of-sight, which represents the impact of SIS errors on code observations. SISURE varies with the location of users, therefore, the Instantaneous SISURE (IURE) and global average SISURE (avgURE) are widely used to describe the statistics of SIS errors and detect the SIS anomaly [2].

Post processing and the real-time estimation are two widely-used methods for SIS anomaly monitoring [6]. The post processing method obtains the SISURE by comparing the broadcast ephemeris and the final precision ephemeris [7]. Cohenour and Kovach used the post-processing method to analyze the anomalous characteristics of Global Positioning System (GPS) broadcast ephemeris [7,8]. However, the final precision ephemeris has the latency of about 15 days, which is not applicable in real-time positioning service. SISURE can be estimated in real time through extracting the other observation error components when given the precise coordinates of users. Gao proposed a real-time estimation method of SISURE through carrier-smoothed-code method with fixed smoothing length [6]. Heng and Gunning optimized the GPS and GLObal NAvigation Satellite System (GLONASS) SIS anomaly detection method by combining the post and real-time estimation methods of SISURE [2,9,10,11,12,13]. Jiang proposed a method to predict the real-time trend of BDS SISURE and optimized the threshold of SISURE anomaly detection based on trend-eliminating SISURE [14]. At present, the real-time estimation method of SISURE is mainly based on either code observation or the smoothed code. Thus, the estimation accuracy of SISURE is limited by the code observation noise. The high-precision SISURE estimation is beneficial for identifying the SIS anomaly in time, which is significant for improving the reliability of real-time positioning. Therefore, it is necessary to optimize the carrier-smoothed-code algorithm to obtain high-precision SISURE.

There are many typical methods, such as Detection, Identification, Adaptation (DIA) and Receiver Autonomous Integrity Monitoring (RAIM), that can be used for anomaly detection [15,16]. Both the DIA and the RAIM can be implemented by comparing the constructed test statistic and the detection threshold obtained from the distribution of the test statistic. The statistical distribution of SISURE for GPS and GLONASS have been extensively studied based on the post processing method, such as Cohenour, Walter and Heng [7,17,18,19]. It has been showed that the SISURE of GPS follows zero-mean Normal distribution, and the GPS User Range Accuracy (URA) can bound the SISURE. For the emerging systems, Chen proposed the calculation method of BDS SISURE [20]. Montenbruck constructed the unified calculation methods of global avgURE for GPS, GLONASS, Galileo and BDS [21,22]. There are few research results on BDS SISURE distribution. In addition, BDS has different constellation configuration, signal system and ephemeris calculation method when compared with GPS [1,23,24]. Therefore, the statistical characteristics of BDS SISURE is normally different from GPS [3,20,21,22]. Although BDS characterizes the accuracy of SISURE by URA contained in the broadcast ephemeris, Wu has shown that the URA broadcasted by BDS is insufficient to ensure the accuracy of SISURE [3]. Therefore, it is necessary to analyze the statistical distribution of BDS SISURE so that the detection threshold of anomaly can be obtained. The proposed method is implemented by comparing the test statistic of IURE and the detection threshold from integrity requirement, which is consistent with the DIA and RAIM. The contribution of the proposed method is to guarantee the SIS anomaly detection power by the innovative modelling and estimation of IURE, so that the zero-mean Normal distribution of estimated IURE can be more solid.

Based on the analysis of SISURE statistical characteristics, we focus on the real-time detection method for BDS SIS anomalies. Firstly, we analyze the IURE calculation method. Based on the statistical distribution of IURE, we establish the IURE model. Secondly, through the constructed Kalman filtering-based carrier-smoothed-code algorithm, we propose the IURE estimation method and derive the threshold for anomaly detection. Finally, the IURE estimation performance is evaluated and discussed. The performance of anomaly detection and its impact on Single Point Positioning (SPP) are verified by observation of ground stations.

## 2. IURE Modeling

**Figure 1:**
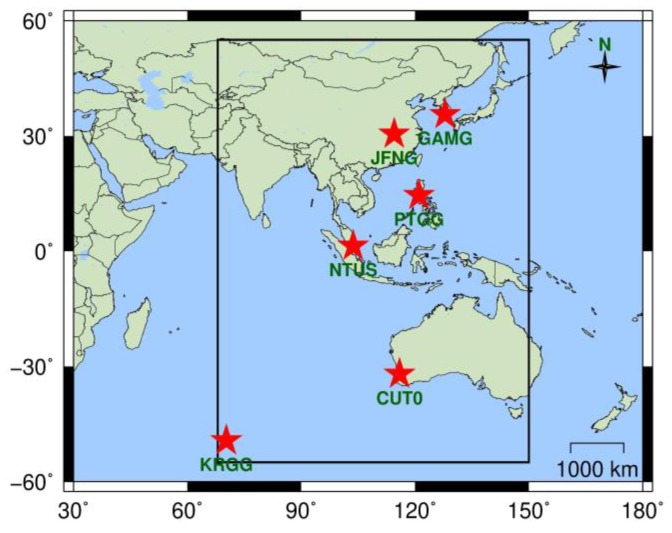
BDS station distribution map. The BDS service area is within the black box. The position of the red star indicates the position of station.

Because the current BDS provides public service area shown by the black box in Figure 1 [1], the IURE is more suitable for anomaly detection than global avgURE. In order to achieve real-time anomaly detection, we construct the IURE model by analyzing IURE statistical characteristics.

### 2.1. IURE Computation

Based on the final precision ephemeris, the SIS errors of broadcast ephemeris can be calculated. Firstly, the time and space references of two types of ephemeris must be coordinated. For the spatial reference, the coordinate system and the center of satellite must be considered [21]. The orbital position coordinate system of BDS broadcast ephemeris is China Geodetic Coordinate System 2000 (CGCS2000). The satellite coordinate obtained from broadcast ephemeris is with respect to Antenna Phase Center (APC) [1]. The orbital position coordinate system of the final precise ephemeris is International Terrestrial Reference Frame 2008 (ITRF2008). The satellite coordinate obtained from final precise ephemeris is based on the Center of Mass (CoM) [3]. Fortunately, the difference between CGCS2000 and ITRF2008 is at centimeter level and can be ignored when calculating SISURE [3,4,21,22]. In order to eliminate the difference between APC and CoM, the antenna Phase Center Offset (PCO) is usually corrected before comparing the broadcast ephemeris with the final precision ephemeris [2,3,21,22].

In order to coordinate the time system, the group-delay and time-reference must be considered. The clock of broadcast ephemeris is measured by using B3I signal, whereas the clock of precision ephemeris is estimated by using the dual-frequency combination of B1I and B2I signals [3,21]. In order to eliminate the bias caused by different measuring methods, the group-delay of satellites should be eliminated:

(1) $$\delta \hat{t}=\delta {\hat{t}}_{brd}-\left(l_{1}\delta t_{TGD1}-l_{2}\delta t_{TGD2}\right)$$

where $l_{1}=\frac{f_{1}^{2}}{f_{1}^{2}-f_{2}^{2}}$, $l_{2}=\frac{f_{2}^{2}}{f_{1}^{2}-f_{2}^{2}}$. $f_{1}$, and $f_{2}$ represent the frequency of BDS B1I and B2I signals separately. $\delta t_{TGD1}$ and $\delta t_{TGD2}$ denote group-delay of B1I and B2I signals, respectively. $\delta {\hat{t}}_{brd}$ and $\delta \hat{t}$ represent original clock derived from the broadcast ephemeris and clock corrected by group-delay, respectively.

Different satellite clock products refer to different time-reference. There is an unignorable bias between two different clock products [21,22]. In order to eliminate the bias, we use the weighted average method to estimate the common bias of all satellites’ clocks:

(2) $$\hat{\mu }\left(k\right)=\frac{1}{\sum_{j=1}^{N}w^{j}}\sum_{j=1}^{N}w^{j}\left[\delta {\hat{t}}^{j}\left(k\right)-\delta t^{j}\left(k\right)\right]$$

where $\hat{\mu }(k)$ is the common bias between two clock products at *k*th epoch, $\delta {\hat{t}}^{j}$ and $\delta t^{j}$ are the satellite clock of broadcast ephemeris and final precision ephemeris for satellite j. *N* is the total number of all valid satellites. $w^{j}$ represents the weight of satellite j when calculating the common bias. To avoid the potential impact of the satellites clock outliers, the $\delta {\hat{t}}^{j}\left(k\right)-\delta t^{j}\left(k\right)$ with a larger residual is weighted toward less contribution by $w^{j}=1/\left|\delta {\hat{t}}^{j}\left(k\right)-\delta t^{j}\left(k\right)-\hat{\mu }\left(k\right)\right|$. Iterations are applied at each epoch until $\hat{\mu }\left(k\right)$ converges to an acceptable level.

The common bias $\hat{\mu }(k)$ is the time-reference bias between two clock products. The clock error of broadcast ephemeris can be obtained by subtracting the time-reference bias from the difference between broadcast ephemeris and final precision ephemeris clock product.

When the time and space reference of the precise and the broadcast ephemeris are consistent, the IURE of broadcast ephemeris can be calculated by taking the final precise ephemeris as true values:

(3) $$IURE^{j}=\left({\hat{X}}^{j}-X^{j}\right)\cdot \frac{X^{j}-X_{r}}{\left\|X^{j}-X_{r}\right\|}-c\left(\delta {\hat{t}}^{j}-\delta t^{j}-\hat{\mu }\left(k\right)\right)$$

where $IURE^{j}$ represents the IURE of satellite j, ${\hat{X}}^{j}$ and $X^{j}$ represent the satellite position calculated by using broadcast ephemeris and final precision ephemeris, respectively. c represents the speed of light. $X_{r}$ represents the position of the station $\left\|x\right\|=\sqrt{x\cdot x^{T}}$.

In order to analyze the distribution of IURE, the outliers of IURE should be eliminated. At present, BDS has not released the relevant indicators of integrity. We assume the URA definition of BDS is similar with GPS [2,3,25]. Therefore, the outlier is eliminated with a threshold of 4.42 × *URAUB*, where *URAUB* indicates the upper boundary of URA.

### 2.2. IURE Model

**Figure 2:**
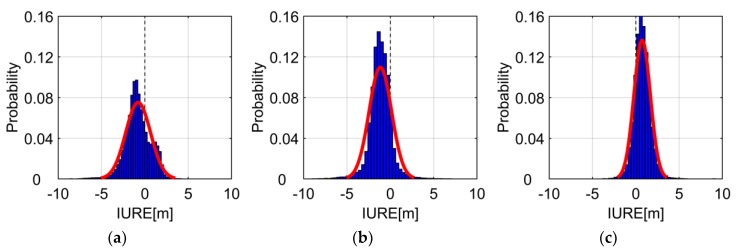
Histogram of IURE at CUT0 station. The blue region is the histogram of IURE and the red line is the fitting curves of Normal distribution Probability Density Function (PDF). (**a**–**c**) are the results of GEosynchronous Orbit (GEO) satellite C01, Inclined Geosynchronous Satellite Orbit (IGSO) satellite C06 and Medium Earth Orbit (MEO) satellite C11, respectively.

The accurate IURE model relies on the statistical characteristics of IURE. It can be seen from (3) that the IURE changes with the coordinates of stations due to the effect of line-of-sight. Due to the limitations of the content, we take “CUT0” station as shown in Figure 1 as an example to analyze the statistical characteristics of IURE. The broadcast ephemeris comes from the “BRDM” products provided by International GNSS Service (IGS) (ftp://cddis.gsfc.nasa.gov/pub/gps/data/campaign/mgex/daily/rinex3/). The final precision ephemeris is provided by WuHan University (WHU) (ftp://igs.ign.fr/pub/igs/products/mgex/). The time range is from February 18, 2013 to June 2, 2018, and the sampling interval is 15 minutes. The broadcast ephemeris-based satellite orbit and clock are interpolated based on BDS interface control document [1]. A total of 185472 samples are collected for the last five years. Other stations have similar results. Only three typical satellites are plotted, and the IURE within ±10 m is shown in Figure 2.

It can be seen from the Figure 2 that the unimodal characteristics of IURE distribution are obvious for MEO and IGSO satellites, but GEO satellites have multiple peaks. In addition, the IURE distributions have skewed bias of −0.78 m, −1.15 m, and 0.73 m, respectively. The skewed bias distribution is caused by a systematic bias originated from satellite clock errors [3]. Therefore, the distribution of IURE does not follow zero-mean normal distribution.

**Figure 3:**
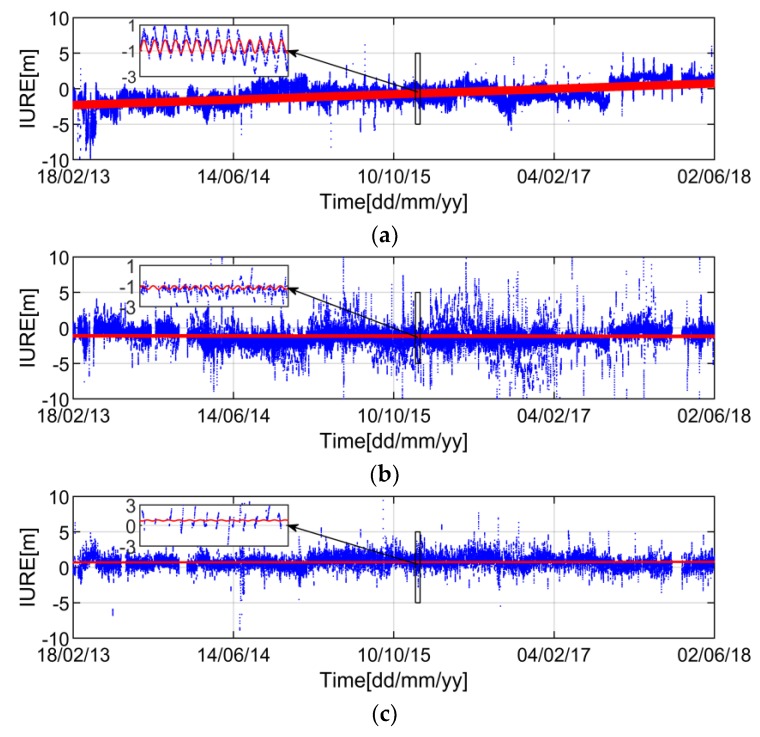
Time series of IURE and $IURE_{trend}$ at CUT0 station. The blue dots and red lines represent the IURE and $IURE_{trend}$, respectively. Each subpanel contains a zoomed-in portion. (**a**–**c**) are the results of GEO satellite C01, IGSO satellite C06 and MEO satellite C11, respectively. [dd/mm/yy] represents [day/month/year].

Based on the time series of IURE from Figure 3, we can find that the IURE presents a certain periodicity. Therefore, we establish the IURE model as:

(4) $$IURE=IURE_{trend}+IURE_{stoc}=p\left(1\right)+p\left(2\right)\Delta t+p\left(3\right)\sin\left(\frac{2pi}{T_{b}}\Delta t\right)+p\left(4\right)\cos\left(\frac{2pi}{T_{b}}\Delta t\right)+IURE_{stoc}$$

where $IURE_{trend}$ and $IURE_{stoc}$ represent the trend term and the random term of IURE. p(1), p(2), p(3) and p(4) represent the parameters of trend term. $\Delta t=\frac{t-t_{0}}{T_{s}}$ represents the normalized time parameters. t, $t_{0}$ and $T_{s}$ represent the observation time, the initial time and the sampling period, respectively. $T_{b}$ is the normalized period, and pi represents the circumference ratio. We make $T_{b}$ equal to a sidereal day. 

As shown in Figure 3, three typical satellites are plotted and the IURE within ±10 m are shown. We can see that each satellite has a systematic bias, which is mainly affected by the clock error [3]. In addition, it can be seen from the zoomed-in portion that the IURE of GEO satellite has obvious periodic feature. The periodic fluctuation is mainly caused by the period of satellite motion [20]. Although the $IURE_{trend}$ depends on the line-of-sight as well as the user location, we can see the long-term stability of $IURE_{trend}$ for the past five years. *IURE_trend_* is a function of time with the coefficients *p*(*i*) (*i* = 1, 2, 3, 4) which can be estimated by the least square method based on the historical IURE data.

**Figure 4:**
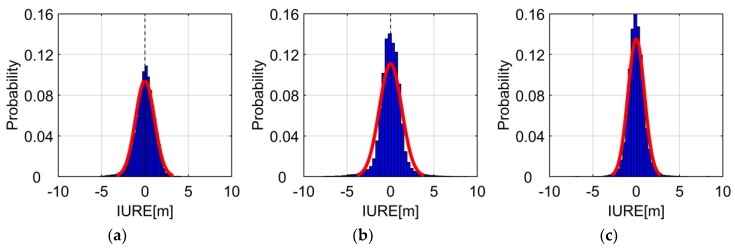
Histogram of $IURE_{stoc}$ at CUT0 station. The blue region is the histogram of $IURE_{stoc}$ and the red line is the fitting curve of a normal distribution PDF. (**a**–**c**) are the results of GEO satellite C01, IGSO satellite C06 and MEO satellite C11, respectively.

Figure 4 plots the histogram of $IURE_{stoc}$ for CUT0 station. The red fitting curve can fit the distribution of IURE. The mean of $IURE_{stoc}$ is very close to 0, especially for GEO satellites, we can see that the multi-peak is eliminated. The distribution of $IURE_{stoc}$ is unimodality and symmetry, which indicates that the $IURE_{stoc}$ can be assumed to follow zero-mean normal distribution. Overall, the IURE can be modelled as the combination of the zero-mean normal distribution with a trend bias.

## 3. IURE Anomaly Detection

Based on the constructed IURE model, we can achieve real-time estimation of IURE. High-precision IURE estimation can be beneficial for not only accurately characterizing the ranging error, but also quickly detecting SIS anomalies with a reasonable threshold. Therefore, we propose a real-time IURE estimation method by using the Kalman filtering-based carrier-smoothed-code to improve the accuracy of IURE estimation. Furthermore, we obtain the threshold to detect SIS anomaly based on the integrity requirement.

### 3.1. Real-Time IURE Estimation

Since the phase observation suffers from the issue of ambiguity resolution, we therefore utilize the code observation for the IURE real-time estimation. The code observation can be modelled as:

(5) $$\left\{ \begin{array}{l} \rho_{1}=r+c\delta t_{u}-c\left(\delta t_{brd}+\delta t_{r}-\delta t_{TGD1}\right)+T+I+\varepsilon_{\rho_{1}}\\ \rho_{2}=r+c\delta t_{u}-c\left(\delta t_{brd}+\delta t_{r}-\delta t_{TGD2}\right)+T+\frac{f_{1}^{2}}{f_{2}^{2}}I+\varepsilon_{\rho_{2}} \end{array}\right.$$

where *r* is the actual distance between the ground station and the satellite. $\delta t_{u}$, $\delta t_{brd}$ and $\delta t_{r}$ represent the clock bias caused by receiver, satellite and relativity, respectively. T denotes tropospheric delay error. $\rho_{i}$ represents the code observation of carrier $f_{i}$. I is the ionospheric delay on carrier $f_{1}$. $\varepsilon_{\rho_{i}}$ is the observed noise of carrier $f_{i}$. In addition, the influence of the earth’s rotation is corrected in the calculation of *r*.

In order to improve the estimation performance of IURE, it is necessary to reduce the effect of various atmospheric errors. For dual-frequency signals, the Ionosphere-Free (IF) combination is used:

(6) $$\rho_{IF}=l_{1}\rho_{1}-l_{2}\rho_{2}=r-c\left(\delta t_{brd}+\delta t_{r}-l_{1}\delta t_{TGD1}+l_{2}\delta t_{TGD2}\right)+c\delta t_{u}+T+\varepsilon_{\rho_{IF}}$$

where $\rho_{IF}$ denotes IF observation. $\varepsilon_{\rho_{IF}}$ is the model error and observed noise of $\rho_{IF}$. In general, we utilize the traditional carrier-smoothed-code to suppress the amplified IF observation noise:

(7) $$\hat{\rho }\left(k\right)=\frac{1}{M}\rho \left(k\right)+\frac{M-1}{M}\left[\hat{\rho }\left(k-1\right)+\phi \left(k\right)-\phi \left(k-1\right)\right]$$

where ρ(k), ϕ(k) and $\hat{\rho }\left(k\right)$ represent the code, carrier phase and carrier-smoothed-code of the satellite at *k*th epoch, respectively. *M* denotes the traditional smoothing length. 

The standard implementation of Local Area Augmentation System (LAAS) recommends the smoothing length is fixed to 100 s for GPS [26]. At present, there is no official recommendation of smoothing length for BDS [1,25,27]. Due to the different signal systems, BDS and GPS are different in terms of code observation multipath and noise [28,29]. Therefore, the smoothing length of GPS may not be suitable for BDS. In addition, the fixed smoothing length cannot adapt to the observation quality of different satellites because BDS has three types of satellites. In order to select the optimal smoothing length, we rearrange (7) to construct the state and measuring model of Kalman filter:

(8) $$\left\{ \begin{array}{l} \hat{\rho }\left(k\right)=\left[1+\frac{\phi \left(k\right)-\phi \left(k-1\right)}{\hat{\rho }\left(k-1\right)}\right]\hat{\rho }\left(k-1\right)+W\left(k\right)\\ \rho \left(k\right)=\hat{\rho }\left(k\right)+V\left(k\right) \end{array}\right.$$

where W(k) and V(k) are the system process noise and observation noise, respectively. The gain K of Kalman filter is the reciprocal of M. The carrier-smoothed-code based on Kalman filter can realize the optimal control of M by using the gain K to suppress the code noise. It should be noted that it has been widely proven that raw BDS code and phase observation follow Gaussian distribution [28,29]. Therefore, we select the traditional linear Kalman filter for the carrier-smoothed-code.

We define $r=\hat{r}-\varepsilon_{e}$, $\delta t=\delta t_{brd}-l_{1}\delta t_{TGD1}+l_{2}\delta t_{TGD2}=\delta \hat{t}-\varepsilon_{c}$, in which $\hat{r}$ represents the range calculated by the broadcast ephemeris. $\varepsilon_{e}$ and $\varepsilon_{c}$ denote the projection of orbit and clock error on the line-of-sight, respectively. Since the IURE can be modeled as $IURE=IURE_{trend}+IURE_{stoc}=\varepsilon_{e}-c\varepsilon_{c}$, the IURE can therefore be calculated as:

(9) $$IURE=\hat{r}-{\hat{\rho }}_{IF}+c\delta t_{u}+T-c\left(\delta \hat{t}+\delta t_{r}\right)+\varepsilon_{{\hat{\rho }}_{IF}}$$

where ${\hat{\rho }}_{IF}$ and $\varepsilon_{{\hat{\rho }}_{IF}}$ are smoothed code and the corresponding noise. $\hat{r}$ can be calculated by the satellite position derived from the broadcast ephemeris and the station position obtained from IGS. ${\hat{\rho }}_{IF}$, $\delta \hat{t}$, and $\delta t_{r}$ can be obtained from the receiver observation file and the broadcast ephemeris. Most of T errors can be eliminated by Saastamoinen model [30]. Therefore, we define:

(10) $$\rho_{cal}=\hat{r}-{\hat{\rho }}_{IF}-c\left(\delta \hat{t}+\delta t_{r}\right)+T$$

The IURE can be computed by combining (9) and (10):

(11) $$IURE=IURE_{trend}+IURE_{stoc}=\rho_{cal}+c\delta t_{u}+\varepsilon_{{\hat{\rho }}_{IF}}$$

It can be found that the receiver clock error $\delta t_{u}$ is unknown at the right side of (11). For any satellite j with the same observation time, we have $IURE^{j}=IURE_{trend}^{j}+IURE_{stoc}^{j}=\rho_{cal}^{j}+c\delta t_{u}+\varepsilon_{{\hat{\rho }}_{IF}}^{j}$. Since the $IURE_{stoc}$ follows the zero-mean normal distribution:

(12) $$\frac{1}{N}\sum_{j=1}^{N}IURE_{stoc}^{j}=\frac{1}{N}\sum_{j=1}^{N}\rho_{cal}^{j}-\frac{1}{N}\sum_{j=1}^{N}IURE_{trend}^{j}+c\delta t_{u}+\frac{1}{N}\sum_{j=1}^{N}\varepsilon_{{\hat{\rho }}_{IF}}^{j}=0$$

where $IURE_{trend}^{j}$ represents the trend term of satellite j that can be derived from historical data. For zero-mean observation noise, it can be assumed that $\frac{1}{N}\sum_{j=1}^{N}\varepsilon_{{\hat{\rho }}_{IF}}^{j}=0$, therefore, the receiver clock can be estimated as:

(13) $$\delta {\hat{t}}_{u}=\frac{1}{cN}\sum_{j=1}^{N}\left(IURE_{trend}^{j}-\rho_{cal}^{j}\right)$$

and then, the IURE can be estimated as:

(14) $$IU\hat{R}E^{j}=\rho_{cal}^{j}+\frac{1}{N}\sum_{j=1}^{N}\left(IURE_{trend}^{j}-\rho_{cal}^{j}\right)$$

Based on the statistical distribution of IURE and the instantaneous observation collected at the station with precise locations, the real-time IURE estimation method can be implemented. Particularly, besides the atmospheric error correction, the receiver clock is estimated and extracted based on the zero-mean normal distribution assumption of *IURE_stoc_*. Regarding to the code observation noise, the smoothing length of carrier-smoothed-code is adjusted in real time by the constructed Kalman filter from (8), which adaptively suppresses the observation noise. Therefore, the estimation performance of IURE can be improved with deliberate observation error correction.

### 3.2. Detection Threshold

Threshold plays a crucial role for the anomaly detection. GPS had provided the SIS integrity definition and requirement in 2008 [31]. The threshold for the anomaly detection can be obtained as 4.42×URAUB:

(15) $$P\left(\left|IURE\right|>4.42\times URAUB\right)<1\times 10^{-5}$$

where $1\times 10^{-5}$ is the integrity requirement given by GPS [31]. At present, although BDS characterizes the accuracy of SISURE by URA contained in the broadcast ephemeris, BDS has not released the definitions of integrity. If (15) is used to determine the threshold, the IURE must follow zero-mean normal distribution. According to the previous analysis, it is found that the statistical distribution of BDS IURE are quite different from zero-mean normal distribution. Therefore, the threshold of 4.42×URAUB is inappropriate for the anomaly detection of BDS IURE. Fortunately, the distribution of $IURE_{stoc}$ can be approximated as zero-mean normal distribution, so the threshold for IURE anomaly detection can be obtained as:

(16) $$P\left(\left|IURE_{stoc}\right|>4.42\times \sigma_{IURE_{stoc}}\right)=P\left(\left|IURE-IURE_{trend}\right|>4.42\times \sigma_{IURE_{stoc}}\right)<1\times 10^{-5}$$

We can take $IURE_{trend}\pm 4.42\times \sigma_{IURE_{stoc}}$ as the threshold to detect BDS IURE anomaly, where $IURE_{trend}$ and $\sigma_{IURE_{stoc}}$ can be derived from the historical data analysis.

Based on above analysis, a potential BDS SIS anomaly will be claimed when an IURE exceeds the threshold and the broadcast ephemeris is flagged as healthy. In general, the real-time detection of SIS anomaly needs to cross check the IURE anomalies of worldwide multiple stations in order to ensure the reliability of SIS anomaly detection. In this contribution, although the detection process of single station is implemented, the detection method can be the foundation for the cross check at multiple stations. 

Based on the zero-mean normal distribution of *IURE_stoc_*, the threshold for BDS IURE anomaly detection is derived from the integrity requirement. It can be anticipated that the performance of SIS anomaly detection will be enhanced.

## 4. Experiments and Discussion

To verify the performance of the proposed IURE estimation method, we analyze the estimation errors of IURE based on raw code observation, denoted as the RCO method, the traditional carrier-smoothed-code, denoted as the TCSC method, and the Kalman filtering-based carrier-smoothed-code, denoted as the KCSC method. Moreover, we use the Root Mean Square (RMS) and STandard Deviation (STD) of the estimation errors to verify the accuracy and reliability of real-time IURE estimation, respectively. In order to demonstrate the edge of threshold derived from the integrity requirement, we analyze the effect of two thresholds on the IURE anomaly detection, i.e., the traditional threshold ±4.42×URAUB, denoted as TTH, and the proposed threshold $IURE_{trend}\pm 4.42\times \sigma_{IURE_{stoc}}$, denoted as PTH. In addition, we take CUT0 and “NNOR” stations provided by IGS as the examples to prove the efficiency of the proposed anomaly detection method by using SPP technology.

### 4.1. IURE Estimation and Analysis

**Figure 5:**
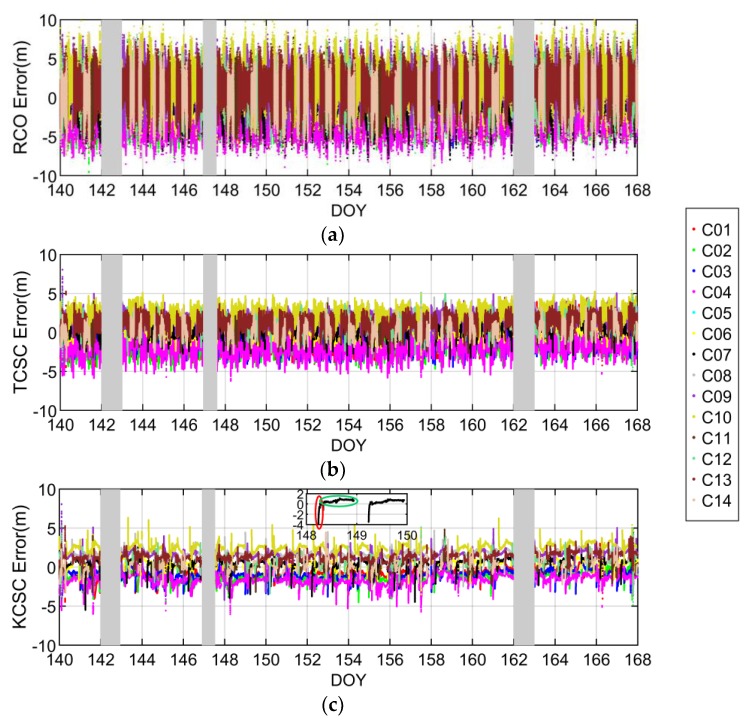
IURE estimation errors of CUT0 station. (**a**–**c**) are the results by using RCO, TCSC and KCSC method, respectively. DOY denotes day of year.

In order to sufficiently evaluate the estimation performance of IURE, we collected raw data from six stations as shown in Figure 1. The data sampling rate is 1 Hz and the experiment time is from 20 May 2018 to 16 June 2018, a total of 2,419,200 samples in 4 weeks. The elevation cutoff is set to 20°. The traditional smoothing length *M* is set to 100s. The observation of stations comes from IGS (ftp://cddis.gsfc.nasa.ov/highrate/2018/) except CUT0 whose observation comes from Curtin GNSS Research Centre (http://saegnss2.curtin.edu.au/ldc/rinex3/daily/2018/). The reference of IURE is calculated by the post processing method. Due to the limitation of real-time data distribution, we use the offline data to simulate the real-time operation. The IURE estimation result of CUT0 station are given in Figure 5.

As observed from Figure 5, there are three parts of data missing as shown in gray area of each subpanel, in which the first and third segments are the absence of the final precise ephemeris, and the second segment is caused by BDS signal interruption. In addition, C05 has been deleted because the frequent signal interruption. Figure 5c contains a zoomed-in portion of C07 satellite. As shown in the red circle of zoomed-in portion, some newly observed satellites have large estimation errors because either low elevation or poor observation quality, and the estimation errors gradually converged when the elevation increases. Moreover, we can find that there is a systematic bias for each satellite as shown in the green circle of zoomed-in portion, which may be caused by the multipath and the unmodelled bias. It can be seen from Figure 5 that the IURE estimation accuracy of KCSC method and TCSC method are better than the RCO method, which illustrate the necessity of code noise suppression. Furthermore, the estimation errors of KCSC method are relatively less than the TSCS method. It reveals the efficiency of utilizing the Kalman filter to determine the optimal smoothing length. The higher IURE estimation accuracy will provide better performance of SIS anomaly detection.

According to the three types of BDS satellites, Table 1 and Table 2 give the numerical statistics of the estimation errors for six stations. Table 1 summarizes the RMS of IURE estimation errors. As seen from Table 1, a minimum RMS of 0.74 m can be obtained from the MEO satellite at JFNG station by using the KCSC method. Moreover, we find that the GEO satellite have the largest RMS of IURE estimation errors for three methods, which can be explained by the lowest precision of final precision ephemeris for GEO satellites compared with IGSO and MEO satellites [32]. Meanwhile, it can be seen that stations at low latitudes can achieve smaller RMS than the high latitudes, which may be because stations at low latitudes can observe more satellites and achieve higher estimation accuracy of receiver clock. Regardless of the satellite types and the location of stations, it can be found that the KCSC method can achieve better estimation accuracy than the RCO method and the TCSC method, which proves the estimation accuracy of real-time IURE is benefit from the Kalman filtering-based carrier-smoothed-code algorithm. Compared with the RCO and TCSC methods, the RMS of IURE estimation errors obtained by the KCSC method is reduced by 17.3% and 8.3% respectively.

The STD of IURE estimation errors is shown in Table 2, from which we find that a minimum STD of 0.39 m can be obtained from the GEO satellite at NTUS station by using the KCSC method. The MEO satellite have the largest STD compared with GEO and IGSO satellites for all methods as shown in Table 2. This mainly because the observation of MEO satellites is discontinuous, and there are more convergence processes. In addition, there is no obvious relationship between STD and station location. Compared with he RCO and TCSC methods, the STD obtained by the KCSC method is the smallest for all satellite types and all sites, which indicates that the proposed algorithm can effectively improve the reliability of real-time IURE estimation. Overall, compared with RCO and TCSC methods, the STD of IURE estimation errors obtained by the KCSC method is reduced by 38.2% and 22.5%, respectively. The IURE estimation accuracy of 1.1 m is sufficient for satisfying the integrity requirement of $1\times 10^{-5}$, which is significant for applying the proposed method for the BDS-based safety-critical services.

### 4.2. Detection Performance of SIS Anomaly Based on the IURE Estimation

We define the SIS anomaly is caused by orbit fault when the contribution of the orbit error to the IURE is greater than the clock error. If the contribution of the clock error to the IURE is greater than the orbit error, we define the anomaly is caused by a clock fault.

**Figure 6:**
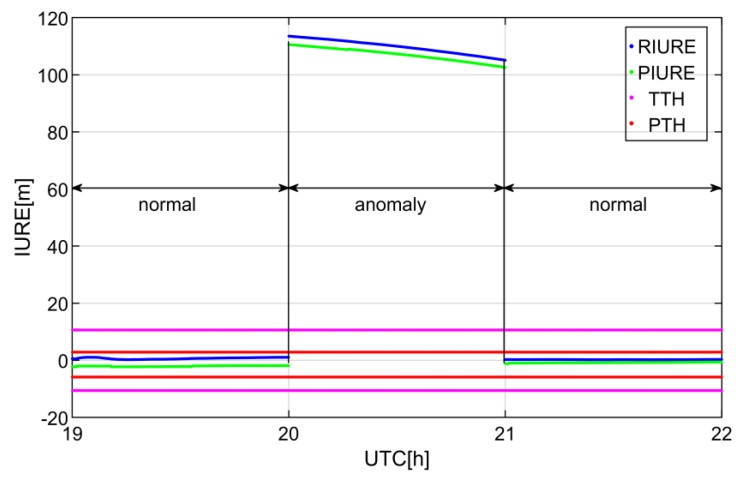
Detection process of SIS anomaly caused by orbit fault on 23 November 2016. The anomaly state indicates that the IURE is larger than the threshold when the broadcast ephemeris is marked as healthy, otherwise it is the normal state. RIURE and PIURE represent the real-time IURE estimation and the reference of IURE respectively. UTC denotes coordinated universal time.

The SIS anomaly detection process of C09 is shown in Figure 6. This SIS anomaly is caused by orbit fault and is detected at CUT0 station. It can be seen from the Figure 6 that the real-time IURE can track the IURE reference. Regardless of detection thresholds, the SIS can be marked as anomaly from the 20th to the 21th hour.

**Figure 7:**
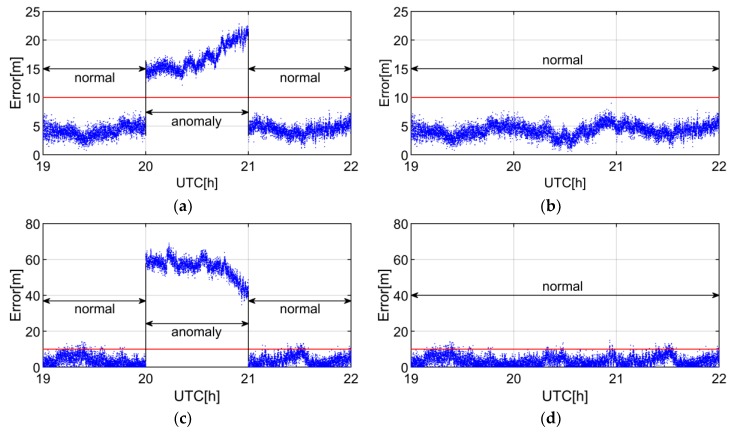
SPP errors of CUT0 station. The blue dots and red lines indicate the positioning errors and the threshold of 10 m. The normal state indicates that the positioning error is consistent with the position accuracy requirement, whereas the anomaly state means the position accuracy requirement cannot be satisfied. (**a**,**c**) represent the horizontal and vertical positioning errors with C09 when the SIS is marked as anomaly, respectively. (**b**,**d**) denote the horizontal and vertical positioning errors without C09 when the SIS is marked as anomaly, respectively. UTC denotes coordinated universal time.

To further verify the efficiency of anomaly detection, we analyze the impact of C09 SIS anomaly on the SPP positioning errors. Figure 7 shows the positioning errors whether or not C09 is engaged in SPP solution from the 20th to the 21th hour. In the normal state of Figure 7c,d, there are several blue dots that exceed the red threshold, but the positioning error still meets the positioning accuracy requirement which demands that the error does not exceed 10 m under the metric of 95% probability [25]. As shown in Figure 7a,c, both the horizontal and vertical errors exceed 10 m when C09 is engaged in the position computation. When the observation of C09 is removed, the positioning error meets the positioning accuracy requirement as shown in Figure 7b,d. Therefore, the SIS anomaly detecting is essential to improve the accuracy and the reliability of positioning.

**Figure 8:**
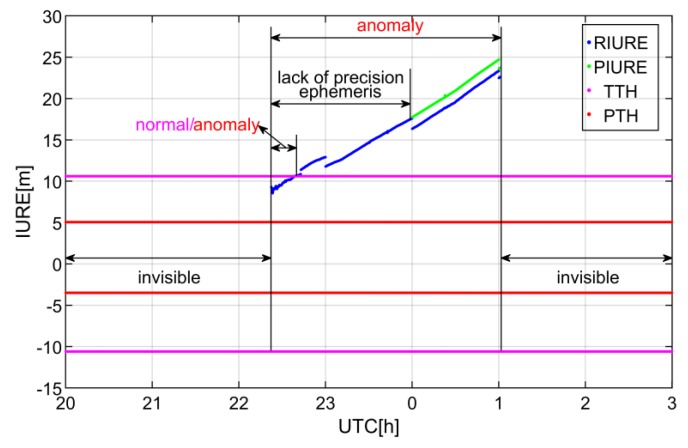
Detection process of SIS anomaly caused by clock fault from the 27th to the 28th day in December, 2016. The invisible state indicates that the receiver has not captured the satellite signal or the elevation is lower than 20°. The state of “lack of precision ephemeris” denotes that the final precise ephemeris is missing. The normal/anomaly state represents that the real-time IURE is greater than PTH but less than TTH when the broadcast ephemeris is marked as healthy. The anomaly state indicates that the real-time IURE is larger than PTH when the broadcast ephemeris is marked as healthy. RIURE and PIURE represent the real-time IURE estimation and the reference of IURE respectively. UTC denotes coordinated universal time.

The SIS anomaly detection process of C11 is shown in Figure 8. The anomaly of C11 is caused by clock fault and is detected at NNOR station. Note that NNOR is located in Australia. The dual frequency code and carrier observations of NNOR are used for SIS anomaly detection. As seen from the Figure 8, the real-time IURE of C11 surpassed PTH and then exceeded TTH over time. On 27 December 2016, the IURE reference is interrupted due to the lack of the final precise ephemeris. This also implies that the real-time estimation of the IURE can effectively compensate for the absence of the final precise ephemeris for the anomaly detection.

**Figure 9:**
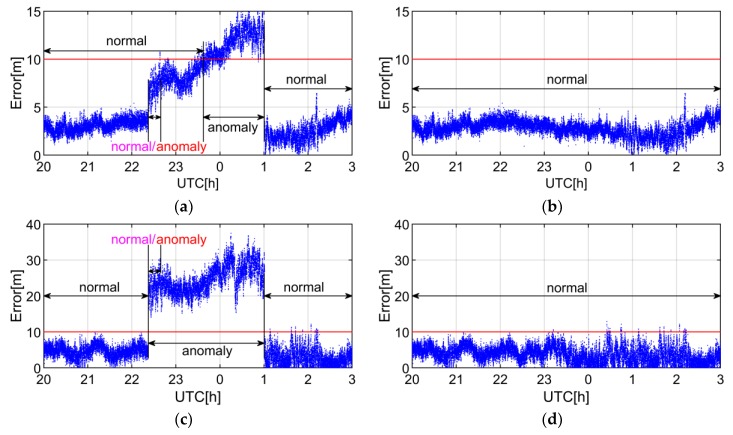
SPP errors of NNOR station. The blue dots and red lines indicate the positioning errors and the threshold of 10 m. The normal state indicates that the positioning error is consistent with the position accuracy requirement, whereas the anomaly state means the position accuracy requirement cannot be satisfied. The normal/anomaly state represents that the real-time IURE is greater than PTH but less than TTH when the broadcast ephemeris is marked as healthy. (**a**,**c**) are the horizontal and vertical positioning errors with C11 when the SIS is marked as anomaly, respectively. (**b**,**d**) denote the horizontal and vertical positioning errors without C11 when the SIS is marked as anomaly, respectively. UTC denotes coordinated universal time.

To further verify the efficiency of anomaly detection, we analyze the impact of C11 SIS anomaly on the SPP positioning errors. Figure 9 shows the positioning errors whether or not C11 is engaged in SPP solution.

As shown in Figure 9a,c, when the IURE is greater than PTH but less than TTH in the normal/anomaly state, the SIS anomaly of C11 causes vertical positioning errors exceeding the position accuracy requirement. If C11 is removed, the positioning accuracy will be constrained to an acceptable level, as shown in Figure 9b,d. It implies that the proposed threshold $IURE_{trend}\pm 4.42\times \sigma_{IURE_{stoc}}$ can better guarantee the accuracy and reliability of positioning than the traditional threshold ±4.42×URAUB, which proves the edge of the proposed threshold based on integrity requirement.

## 5. Conclusions

Through the post-processing of the BDS data collected from 2013 to 2018, we have explored the statistical distribution of IURE. It is found that the PCO should be corrected with different correction products at different stages. Furthermore, it has been shown the IURE does not follow zero-mean normal distribution, but the IURE can be modelled as the combination of the zero-mean normal distribution with a trend bias. With the atmospheric error correction and receiver clock estimation, we develop the IURE estimation method by using the Kalman filtering-based carrier-smoothed-code algorithm in order to suppress the observation noise adaptively. Meanwhile, we obtain the threshold derived from the integrity requirement of 1 × 10^−5^ to detect IURE anomaly. Based on the experiment of six stations, the IURE estimation results has revealed that the RMS and the STD of estimation errors can achieve 1.1 m and 0.55 m, respectively. Compared with the RCO and TCSC methods, the proposed IURE estimation method can obtain the best IURE estimation accuracy regardless of satellite types and locations. Compared with the RCO and TCSC methods, the proposed method can improve the IURE estimate accuracy by 17.3% and 8.3% respectively, the STD of IURE estimation errors obtained by the proposed method is reduced by 38.2% and 22.5% respectively. The test results of CUT0 and NNOR stations show that the proposed detection threshold can provide better accuracy and reliability of user positioning than the traditional detection threshold. Meanwhile, it has been demonstrated that the proposed SIS anomaly detection method can effectively identify the SIS anomalies caused by either orbit faults or clock faults based on the SPP result analysis. Note that the SIS anomalies detected in this contribution are obtained based on the observation of single station. More reliable SIS anomaly detection requires cross-checking among worldwide multiple stations, which will be our future work topic.

## Figures and Tables

**Table 1:** RMS of estimation errors for IURE (Unit: m). “ALL” and “AVG” represent the mean of RMS for all satellites and all stations, respectively.

Type	Method	CUT0	GAMG	JFNG	KRGG	NTUS	PTGG	AVG
GEO	RCO	1.79	1.51	1.32	1.72	1.42	1.09	1.47
	TCSC	1.64	1.45	1.17	1.72	1.42	0.98	1.40
	KCSC	1.31	1.15	1.08	1.72	1.37	0.95	1.26
IGSO	RCO	1.84	1.42	1.49	1.18	1.08	1.37	1.40
	TCSC	1.57	1.27	1.34	1.15	1.07	1.21	1.27
	KCSC	1.45	1.14	1.28	1.11	1.00	1.17	1.19
MEO	RCO	1.56	1.31	1.10	1.00	0.99	1.02	1.16
	TCSC	1.16	1.03	0.84	0.97	0.98	0.80	0.96
	KCSC	0.96	0.87	0.74	0.90	0.89	0.75	0.85
ALL	RCO	1.74	1.41	1.31	1.21	1.15	1.18	1.33
	TCSC	1.47	1.25	1.13	1.19	1.15	1.01	1.20
	KCSC	1.26	1.06	1.05	1.15	1.08	0.97	1.10

**Table 2:** STD of estimation errors for IURE (Unit: m). “ALL” and “AVG” represent the mean of STD for all satellites and all stations, respectively.

Type	Method	CUT0	GAMG	JFNG	KRGG	NTUS	PTGG	AVG
GEO	RCO	1.35	0.99	0.83	0.82	0.42	0.73	0.86
	TCSC	1.15	0.90	0.58	0.81	0.42	0.55	0.74
	KCSC	0.63	0.50	0.45	0.78	0.39	0.52	0.55
IGSO	RCO	1.15	0.89	0.87	0.73	0.56	0.90	0.85
	TCSC	0.71	0.65	0.61	0.68	0.55	0.65	0.64
	KCSC	0.45	0.48	0.50	0.65	0.40	0.59	0.51
MEO	RCO	1.33	1.09	1.00	0.82	0.67	0.97	0.98
	TCSC	0.85	0.77	0.72	0.78	0.67	0.75	0.76
	KCSC	0.59	0.61	0.61	0.67	0.53	0.69	0.61
ALL	RCO	1.27	0.98	0.90	0.78	0.55	0.87	0.89
	TCSC	0.89	0.77	0.64	0.74	0.55	0.65	0.71
	KCSC	0.55	0.53	0.52	0.68	0.44	0.60	0.55
